# Impact of Resolved Preformed, Persistent Preformed, and De Novo Anti-HLA Donor-Specific Antibodies in Kidney Transplant Recipients on Long-Term Renal Graft Outcomes

**DOI:** 10.3390/jcm12103361

**Published:** 2023-05-09

**Authors:** Michal Gniewkiewicz, Katarzyna Czerwinska, Katarzyna Zielniok, Magdalena Durlik

**Affiliations:** 1 Department of Transplantation Medicine, Nephrology and Internal Diseases, Medical University of Warsaw, Nowogrodzka 59, 02-006 Warsaw, Poland; 2 Department of Clinical Immunology, Medical University of Warsaw, Nowogrodzka 59, 02-006 Warsaw, Poland

**Keywords:** resolved preformed DSAs, persistent preformed DSAs, de novo DSAs

## Abstract

The post-transplant evolution of antihuman leukocyte antigen donor-specific antibodies (anti-HLA DSAs) includes three clinical patterns: resolved preformed DSAs, persistent preformed DSAs, and de novo DSAs. The aim of this retrospective study was to analyze the impact of resolved preformed, persistent preformed, and de novo anti-HLA-A, -B, and -DR DSAs in kidney transplant recipients on long-term renal allograft outcomes. This is a post hoc analysis of the study conducted in our transplant center. One hundred eight kidney transplant recipients were included in the study. Patients were followed for a minimum of 24 months after allograft biopsy, which was performed 3 to 24 months after kidney transplantation. The identification of persistent preformed DSAs at the time of biopsy was the most significant predictor of the combined endpoint of the study (>30% decline in estimated glomerular filtration rate or death-censored graft loss; HR = 5.96, 95% CI 2.041–17.431, *p* = 0.0011), followed by the occurrence of de novo DSAs (HR = 4.48, 95% CI 1.483–13.520, *p* = 0.0079). No increased risk was observed in patients with resolved preformed DSAs (HR = 1.10, 95% CI 0.139–8.676, *p* = 0.9305). Patients with resolved preformed DSAs have similar graft prognoses as patients without DSAs, therefore, the persistence of preformed DSAs and development of de novo DSAs are associated with inferior long-term allograft outcomes.

## 1. Introduction

The presence of donor-specific antibodies (DSAs) directed against human leukocyte antigens (HLA) is a risk factor for antibody-mediated rejection (ABMR) and allograft loss in kidney transplant (KTx) recipients [1]. Over the last decade, the identification of anti-HLA DSAs and their characteristics has improved due to the use of solid-phase assay technology, particularly single antigen bead (SAB) testing [2]. In addition, the destructive effect of anti-HLA DSAs is determined by antibody characteristics including class, specificity, mean fluorescent intensity (MFI), complement-biding ability, and IgG subclass [3,4].

DSAs may occur before transplantation (preformed) or arise de novo after transplantation. Alloimmunization before KTx can be caused by pregnancy, blood transfusion, or prior transplant [5,6]. The development of de novo DSAs is associated with several clinical events such as pregnancy, blood transfusion, minimization of immunosuppression, nonadherence, and implementation of a homograft [7,8,9]. Regarding the post-transplant evolution of DSAs, there are three identified clinical patterns: persistent preformed DSAs, resolved preformed DSAs, and de novo DSAs [10]. Patients with persistent preformed DSAs are at a higher risk of ABMR or kidney allograft loss compared to patients with resolved or no preformed DSAs. Factors associated with the persistence of DSAs after transplantation are class II, high MFI, and complement-binding ability [10,11]. However, independently of the evolution status after KTx, preformed DSAs (even with low MFI values) are an important risk factor for decreased graft survival [12]. Patients with identified circulating de novo DSAs display a significantly increased risk of chronic ABMR and renal allograft loss compared to patients with preexisting DSAs [13,14]. Regardless of the preformed/de novo status, the presence of DSAs corresponds with a higher incidence of diagnosis of subclinical ABMR [15,16]. DSA positivity after KTx is associated with increased ABMR-related gene expression, even in biopsy samples with no molecular or histologic rejection [17].

In solid-organ transplantation, the monitoring of anti-HLA DSAs is currently under investigation. Recently, clinical recommendations for the post-transplant assessment of anti-HLA DSAs were published [18]. Nevertheless, there are some deficits in the existing knowledge that should be addressed. There is a need for a better understanding of the clinical role of persistent preformed anti-HLA DSAs and to investigate factors that lead to the clearance of preformed anti-HLA DSAs after transplantation [11]. As the evolution of DSAs after KTx may modify the outcomes, therapeutic interventions could be implemented [18].

The aim of this retrospective study was to analyze the impact of resolved preformed, persistent preformed, and de novo anti-HLA-A, -B, and -DR DSAs in kidney transplant recipients with long-term renal allograft outcomes.

## 2. Materials and Methods

### 2.1. Study Design and Data Collection

This is a post hoc analysis of the study conducted in our transplant center [19]. All 108 consecutive kidney transplant recipients from brain-dead deceased donors from the mentioned study were included in this retrospective study. All patients underwent an ultrasound-guided renal allograft biopsy between 2018 and 2020, 3 to 24 months after KTx, and were followed for a minimum of 24 months after the biopsy (except in cases of death or graft failure).

Patients were divided post hoc into four groups according to their DSA status at the time of biopsy: no anti-HLA DSAs, resolved preformed anti-HLA DSAs, persistent preformed anti-HLA DSAs, and de novo anti-HLA DSAs.

All transplantations were performed with negative complement-dependent cytotoxicity crossmatch. Donor–recipient pairs were ABO blood group compatible. All patients were of white ethnicity. None of the patients received a desensitization protocol before KTx. The decision of whether and what induction therapy (basiliximab or anti-thymocyte globulin (ATG)) to administer was made individually for each patient after the assessment of their immunological risk. Mostly, patients with a high immunological risk received ATG while patients with an intermediate immunological risk received basiliximab. In all patients, the maintenance immunosuppressive regimen consisted of the standard of care agents: a calcineurin inhibitor (tacrolimus or cyclosporine) with mycophenolate mofetil and prednisone. Only two patients were treated with cyclosporine due to tacrolimus intolerance. Biopsies were assessed using Banff classification criteria [20].

At the time of biopsy, serum samples were obtained from all the patients and stored for further analysis. Sera were tested for the presence of anti-HLA DSAs and their characteristics including class, specificity, MFI, C1q-binding capacity, and IgG subclass according to the protocol described previously [19]. Clinical and laboratory data including the DSA status of the patients before KTx were retrospectively extracted from the medical records. All patients were tested for anti-HLA DSAs by SAB assay before transplantation. Antibodies with MFI values above 500 were determined as positive. The identification of donor HLA antibody specificity was limited to HLA-A, HLA-B, and HLA-DR. Donor typing was only available for HLA-A, HLA-B, and HLA-DR; the cross-reactive epitope groups were not considered. The resolved preformed anti-HLA DSAs were defined as antibodies detected before KTx but not detected at the time of the kidney transplant biopsy procedure.

The primary outcome of the study was defined as a permanent 30% decline (two consecutive laboratory assessments ≥ 3 months apart) in the estimated glomerular filtration rate (eGFR) compared to the baseline at the time of biopsy or death-censored graft loss (need for dialysis or retransplantation). The Chronic Kidney Disease Epidemiology Collaboration 2009 (CKD-EPI) creatinine equation was used to determine the eGFR.

Approval of the study was obtained from the Medical University of Warsaw medical ethics committee (Warsaw, Poland). The study was performed according to the principles expressed in the Declaration of Helsinki. All patients provided informed written consent.

### 2.2. Statistical Analysis

Statistical analysis was conducted using R software. The Shapiro–Wilk test was used to assess the normality of distribution. Continuous variables were presented as the means with standard deviation (SD) or medians with quartiles 1 and 3 (Q1–Q3) as appropriate. Categorical variables are presented as frequencies and percentages. Differences between continuous variables were assessed using the *t*-test, Mann–Whitney U test, and one-way analysis of variance (F-test or Kruskal–Wallis test), depending on the number and distribution of the compared variables. The Chi-square test was used to compare categorical variables. The Yates continuity correction was used when the frequency of events was low (<5). Logistic regression was performed to identify variables associated with DSA’s status. Event-free survival was assessed using the Kaplan–Meier method and log-rank test. Univariate and multivariate Cox proportional hazards models were used to quantify the hazard ratios for the study outcome. A backward stepwise elimination method was applied. A *p*-value < 0.05 was considered statistically significant.

## 3. Results

### 3.1. Demographic and Clinical Characteristics

A total of 108 consecutive patients who received kidney transplants from brain-dead deceased donors and underwent renal allograft biopsy 3 to 24 months post-KTx were included in the study. Based on the DSA status at the time of biopsy, four groups were identified: no anti-HLA DSAs (N = 80), resolved preformed anti-HLA DSAs (N = 9), persistent preformed anti-HLA DSAs (N = 9), and de novo anti-HLA DSAs (N = 10). The demographic and clinical characteristics of the groups are provided in Table 1. The groups did not differ in age, sex, body mass index, type of renal replacement therapy, cause of end-stage renal disease, age and sex of the donor, duration of cold ischemia, or number of HLA mismatches. Moreover, no statistical difference was found in terms of clinical characteristics such as the use of tacrolimus versus cyclosporine for maintenance immunosuppression, eGFR and proteinuria at biopsy, protocol biopsy, time from KTx to biopsy procedure, and C4d deposition in biopsy specimens. There was a statistical difference regarding the history of previous renal transplantation, induction of immunosuppression, panel-reactive antibodies, and diagnosis of ABMR at biopsy. The prior KTxs had 2.5% of patients without DSAs, 22.2% of patients with resolved preformed DSAs, 77.8% of patients with persistent preformed DSAs, and 80% of patients with de novo DSAs. The transplant procedure was performed without the induction of immunosuppression in 85% of patients without DSA. In patients with preformed DSAs (resolved and persistent), the induction protocol with anti-thymocyte globulin was the most popular. Half of the patients with de novo DSAs received basiliximab as an induction of immunosuppression. The panel-reactive antibody >5% was the most common in patients with persistent preformed anti-HLA DSAs (55.6%). ABMR was diagnosed in 2.5% of patients without DSAs and in 22.2% and 20% of KTx recipients with preformed and de novo DSAs, respectively.

### 3.2. Characteristics of Anti-HLA DSAs before and after Transplantation

Table 2 depicts the characteristics of anti-HLA DSAs before transplantation. No significant difference was shown between the number, HLA class specificity, and MFI of resolved and persistent anti-HLA DSAs. Nevertheless, the median MFI level of resolved preformed DSAs was lower compared to the median MFI level of persistent preformed DSAs (983 vs. 1905, not statistically significant).

Table 3 shows the characteristics of anti-HLA DSAs at the time of biopsy. There was no significant difference between the persistent preformed and de novo anti-HLA DSAs regarding the number, HLA class specificity, MFI, C1q-binding capacity, and IgG subclasses. However, the median MFI level of persistent preformed anti-HLA DSAs was higher in comparison with de novo anti-HLA DSAs (3843 vs. 1693, not statistically significant).

### 3.3. Clinical Outcomes

All patients were followed for a minimum of 24 months after the biopsy procedure. The clinical outcomes are shown in Table 4. The combined endpoint of the study (>30% decline in eGFR or death-censored graft loss) was reached by 66.7% of patients with persistent preformed DSAs, 50% of patients with de novo DSAs, 11.1% of patients with resolved preformed DSAs, and 10% of patients without DSAs (*p* < 0.0001). Two patients (22.2%) with persistent preformed DSAs experienced graft loss. As many as 42.9% of patients with persistent preformed DSAs had proteinuria at the end of follow-up, ≥50 mg/dL (*p* = 0.0001). No statistical difference between the groups was identified regarding the time of follow-up after biopsy, median proteinuria at the end of follow-up, time from biopsy to the primary outcome, and mortality.

### 3.4. Survival Analysis

**Figure 1:**
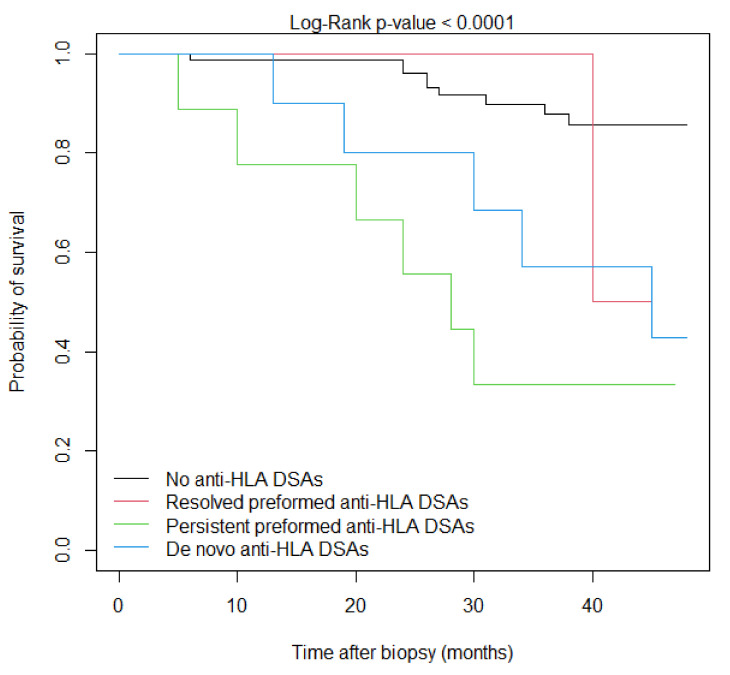
Event-free Kaplan–Meier survival curves according to anti-HLA DSA status at the time of biopsy.

The event-free survival according to DSA status is shown in Figure 1 and the univariate Cox analysis is demonstrated in Table 5. Compared to patients without anti-HLA DSAs, the persistence of preformed anti-HLA DSAs after KTx and development of de novo anti-HLA DSAs at the time of biopsy were significantly associated with inferior survival (*p* < 0.0001 and *p* = 0.0090, respectively). However, there was no statistical difference regarding the combined endpoint survival between patients with resolved preformed anti-HLA DSAs after transplantation and patients without anti-HLA DSAs (*p* = 0.918).

The multivariate Cox regression model for the risk of the combined endpoint is depicted in Table 6. In this model, the identification of persistent preformed anti-HLA DSAs at the time of biopsy is the most significant predictor of inferior graft outcomes (HR = 5.96, *p* = 0.0011), followed by the occurrence of de novo anti-HLA DSAs (HR = 4.48, *p* = 0.0079), proteinuria at biopsy ≥ 50 mg/dL (HR = 1.02, *p* = 0.0428), and increased donor’s age, with borderline statistical significance (HR = 1.03, *p* = 0.0530).

### 3.5. Factors Associated with DSA Status

Multivariate logistic regression analysis revealed several clinical, independent variables significant with DSA status (Table 7). The use of anti-thymocyte globulin therapy was a risk factor for resolved preformed anti-HLA DSAs. The persistence of preformed anti-HLA DSAs was associated with previous transplantation and the increased age of the donor. Moreover, a prior transplant was identified to be a risk factor for the development of de novo anti-HLA DSAs.

## 4. Discussion

In the study, the clinical impact of the evolution of anti-HLA DSAs in kidney transplant recipients was assessed. In recent years, transplant matching has evolved due to the development of new technologies. Moreover, the characteristics of donors and recipients have changed, which alters KTx outcomes [21]. Currently, in most transplant centers, histocompatibility testing for solid organ transplantation includes ABO blood group compatibility, HLA donor–recipient matching, and crossmatching testing [22]. Anti-HLA antibody screening is crucial for assessing the immunological risk in kidney transplant recipients. However, the interpretation of DSAs should be conducted with great care; it demands an understanding of the complexity of antibodies and the technical aspects of detection assays [23].

In the cohort, 50% of patients with preformed anti-HLA DSAs cleared their antibodies after transplantation. None of the patients received desensitization treatment. This is directly in line with previous findings by Sanev et al. In their large study, which included 924 kidney transplant recipients, they also demonstrated that the persistence of DSAs is associated with higher MFI values and antibodies directed against HLA class II [10]. However, this is not shown in our analysis, probably due to the smaller number of patients.

Our results present that KTx recipients with resolved preformed anti-HLA DSAs have similar renal allograft outcomes regarding the eGFR and graft survival compared to patients without DSAs. Overall, these findings are in accordance with the results reported by other researchers [10,24,25]. The mechanism of clearance of DSAs after transplant without desensitization therapy is not fully explained. It could be hypothesized that an immunosuppressive regimen used after transplantation could decrease the production of weak antibodies [10]. In our study, the clearance of DSAs was associated with the use of ATG. It could be speculated that some preformed DSAs could be clinically irrelevant or in some way may be influenced by ATG. Another circumstance that potentially drives DSA disappearance is the development of graft accommodation, but this process is still under investigation [26].

The detection of persistent preformed anti-HLA DSAs at the time of biopsy and the identification of de novo anti-HLA DSAs are the main independent predictors of worse graft outcomes, defined as a 30% sustained decline of the GFR or graft failure. No significant difference between the characteristics of these antibodies was found. This might imply that current, circulating DSAs are more essential in the prediction of the renal allograft compared to resolved DSAs. A similar conclusion was reached in studies involving not only adult renal transplant recipients but also patients after other solid organ transplantation such as heart, lung, or intestine [27,28,29,30]. A history of previous transplantation is independently associated with the persistence of preformed DSAs. This is consistent with what has been found by Caillard et al. [24]. In addition, the persistence of DSAs was associated with increased donor age. It is difficult to explain such results, but it should be mentioned that the donor’s age was also determined to be a risk factor for overall graft failure [31]. In our model, prior transplantation is also associated with the development of de novo DSAs. Similar findings were reported before in the liver transplant recipients cohort [32]. These results could suggest that in the case of kidney retransplantation, immunological status and histocompatibility in an individual patient need to be carefully assessed.

Our findings highlight the clinical impact of the clearance of anti-HLA DSAs after renal transplantation. This is particularly important within the context of emerging new desensitization therapies [33,34]. However, factors leading to the clearance or persistence of preformed anti-HLA DSAs should be further investigated. In clinical practice, in kidney transplant recipients, therapeutic decisions should be based on the detected, circulating anti-HLA DSAs rather than considering pre-transplant antibodies. Nevertheless, there is a fundamental need for more studies assessing post-transplant DSA positivity without allograft dysfunction with reference to the utility of kidney allograft biopsies or potential noninvasive biomarkers such as donor-derived cell-free DNA [18].

One of the limitations of the present study is the post hoc, retrospective nature of the analysis. In addition, a relatively modest number of patients had been identified with DSAs in this single-center investigation. The characteristics of pre-transplant antibodies such as C1q-binding capacity and IgG subclasses were not assessed. Donor typing did not include HLA-C, -DP, and -DQ, therefore, antibodies against these antigens were not analyzed and the ABMR risk could not be assessed precisely. The cross-reactive epitope groups were not considered. Patients were tested for post-transplant DSAs only at the time of kidney allograft biopsy, which was performed 3 to 24 months after the transplant. Follow-up biopsies are not available. The use of different agents in the induction of immunosuppressive therapy was inevitable.

To conclude, kidney transplant recipients with resolved preformed anti-HLA DSAs have similar graft prognoses as patients without DSAs. The persistence of preformed anti-HLA DSAs after kidney transplantation and the occurrence of de novo anti-HLA DSAs are independent predictors for inferior long-term allograft outcomes. Candidates for kidney retransplantation should undergo a cautious immunological risk assessment. These findings are valuable in light of the development of new desensitization protocols. Although transplant immunology evolves rapidly, there are many gaps in the current knowledge and further studies are needed to answer clinical questions.

## Figures and Tables

**Table 1:** Characteristics of the groups.

	No Anti-HLA DSAs (N = 80)	Resolved Preformed Anti-HLA DSAs (N = 9)	Persistent Preformed Anti-HLA DSAs (N = 9)	De Novo Anti-HLA DSAs (N = 10)	*p*-Value
Recipient characteristic					
Age at biopsy, years, median (Q1–Q3)	45.0 (38.0–62.0)	49.0 (45.0–54.0)	54.0 (44.0–61.0)	51.0 (45.3–55.0)	0.9637
Male, n (%)	53 (66.3)	5 (55.6)	4 (44.4)	7 (70.0)	0.5517
Body mass index at biopsy, kg/m^2^, mean ± SD	24.9 ± 4.03	26.6 ± 3.15	24.2 ± 2.82	24.3 ± 3.56	0.1589
Previous transplantation, n (%)	2 (2.5)	2 (22.2)	7 (77.8)	8 (80.0)	<0.0001
Renal replacement therapy, n (%)					0.9640
Pre-emptive transplantation	10 (12.5)	1 (11.1)	0	1 (10.0)	
Hemodialysis	60 (75.0)	7 (77.8)	8 (88.9)	8 (80.0)	
Peritoneal dialysis	10 (12.5)	1 (11.1)	1 (11.1)	1 (10.0)	
Cause of ESRD, n (%)					0.6475
Glomerulonephritis	31 (38.8)	5 (55.6)	5 (55.6)	7 (70.0)	
ADPKD	13 (16.3)	2 (22.2)	2 (22.2)	2 (20.0)	
Diabetes	14 (17.5)	0	1 (11.1)	1 (10.0)	
Congenital anomaly	4 (5.0)	1 (11.1)	0	0	
Other	18 (22.4)	1 (11.1)	1 (11.1)	0	
Diabetes, n (%)	24 (30.0)	2 (22.2)	2 (22.2)	3 (30.0)	0.9311
Donor characteristics					
Age, years, mean ± SD	44.8 ± 15.04	46.9 ± 14.07	57.9 ± 9.24	46.7 ± 15.44	0.1306
Male, n (%)	53 (66.3)	5 (55.6)	5 (55.6)	5 (50.0)	0.6789
Transplant characteristics					
Cold ischemia time, minutes, mean ± SD	1239 ± 607.1	1242 ± 450.9	1271 ± 390.3	1562 ± 753.3	0.4476
Induction therapy, n (%)					<0.0001
None	68 (85.0)	2 (22.2)	1 (11.1)	4 (40.0)	
Basiliximab	8 (10.0)	2 (22.2)	3 (33.3)	5 (50.0)	
ATG	4 (5.0)	5 (55.6)	5 (55.6)	1 (10.0)	
HLA mismatches, median, (Q1–Q3)					
A	1.0 (1.0–2.0)	1.0 (1.0–1.0)	1.0 (1.0–1.0)	1.0 (1.0–1.8)	0.3588
B	1.0 (1.0–2.0)	1.0 (1.0–2.0)	1.0 (1.0–2.0)	1.0 (1.0–2.0)	0.5407
DR	1.0 (0.8–1.0)	1.0 (1.0–1.0)	1.0 (1.0–1.0)	1.0 (1.0–1.0)	0.3914
Total	3.0 (3.0–4.0)	3.0 (3.0–4.0)	3.0 (3.0–4.0)	4.0 (3.0–4.0)	0.7966
Panel-reactive antibody >5%, n (%)	7 (8.8)	1 (11.1)	5 (55.6)	2 (20.0)	0.0017
Panel-reactive antibody, median (Q1–Q3)	0 (0–0)	0 (0–0)	57 (0–33)	0 (0–0)	0.0015
Clinical characteristics					
Immunosuppression, n (%)					0.2455
Tacrolimus	79 (98.7)	9 (100.0)	9 (100.0)	9 (90.0)	
Cyclosporine	1 (1.3)	0	0	1 (10.0)	
eGFR at biopsy, mL/min/1.73 m^2^, mean ± SD	54.4 ± 21.78	56.8 ± 16.47	40.9 ± 16.23	51.1 ± 17.39	0.2406
Proteinuria at biopsy ≥50 mg/dL, n (%)	10 (12.5)	1 (11.1)	3 (33.3)	2 (20.0)	0.3770
Proteinuria at biopsy, median (Q1–Q3)	0 (0–10)	0 (0–0)	0 (0–50)	5 (0–10)	0.4762
Protocol biopsy n (%)	45 (56.3)	7 (77.8)	5 (55.6)	4 (40.0)	0.4284
Time from transplantation to biopsy, months, median (Q1–Q3)	5 (3–12)	4 (3–12)	4 (3–7)	4 (3–11)	0.8841
ABMR at the time of biopsy, n (%)	3 (3.8)	2 (22.2)	2 (22.2)	2 (20.0)	0.0363
C4d in biopsy, n (%)	10 (12.5)	0	2 (22.2)	2 (20.0)	0.4792

Abbreviations: HLA—human leukocyte antigen; DSAs—donor-specific antibodies; Q1–Q3—quartile 1–3; SD—standard deviation; ESRD—end-stage renal disease; ADPKD—autosomal dominant polycystic kidney disease; ATG—anti-thymocyte globulin; eGFR—estimated glomerular filtration rate; and ABMR—antibody-mediated rejection.

**Table 2:** Characteristics of anti-HLA DSAs before transplantation.

	Resolved Preformed Anti-HLA DSAs (N = 9)	Persistent Preformed Anti-HLA DSAs (N = 9)	*p*-Value
All anti-HLA DSAs			
Number, median (Q1–Q3)	1.0 (1.0–1.0)	1 (1.0–2.0)	0.5008
HLA class specificity, n (%)			0.5796
I	6 (66.7)	5 (55.6)	
II	3 (33.3)	3 (33.3)	
I + II	0	1 (11.1)	
DSAs with the highest MFI			
HLA class specificity, n (%)			1
I	6 (66.7)	5 (55.6)	
II	3 (33.3)	4 (44.4)	
MFI, median (Q1–Q3)	983 (786–3110)	1905 (1381–2889)	0.4363

Abbreviations: HLA—human leukocyte antigen; DSAs—donor-specific antibodies; Q1–Q3—quartile 1–3; SD—standard deviation; and MFI—mean fluorescent intensity.

**Table 3:** Characteristics of anti-HLA DSAs after transplantation.

	Persistent Preformed Anti-HLA DSAs (N = 9)	De Novo Anti-HLA DSAs (N = 10)	*p*-Value
All anti-HLA DSAs			
Number, median (Q1–Q3)	1.0 (1.0–1.0)	1.0 (1.0–1.0)	0.1457
HLA class specificity, n (%)			0.1572
I	4 (44.5)	3 (30.0)	
II	3 (33.3)	7 (70.0)	
I + II	2 (22.2)	0	
DSAs with the highest MFI			
HLA class specificity, n (%)			0.5085
I	5 (55.5)	3 (30.0)	
II	4 (44.5)	7 (70.0)	
MFI, median (Q1–Q3)	3843 (2900–6206)	1693 (903–3832)	0.1564
C1q binding, n (%)	6 (66.7)	4 (40.0)	0.4825
IgG subclasses, n (%)			
IgG1	7 (77.8)	7 (70.0)	1
IgG2	1 (11.1)	1 (10.0)	1
IgG3	3 (33.3)	4 (40.0)	1
IgG4	1 (11.1)	3 (30.0)	0.6564

Abbreviations: HLA—human leukocyte antigen; DSAs—donor-specific antibodies; Q1–Q3—quartile 1–3; and MFI—mean fluorescent intensity.

**Table 4:** Clinical outcomes.

	No Anti-HLA DSAs (N = 80)	Resolved Preformed Anti-HLA DSAs (N = 9)	Persistent Preformed Anti-HLA DSAs (N = 9)	De Novo Anti-HLA DSAs (N = 10)	*p*-Value
Follow-up after biopsy, months, median (Q1–Q3)	38.5 (28.0–44.3)	33.0 (29.0–39.0)	45.0 (41.0–47.0)	41.5 (36.3–46.5)	0.1969
eGFR at the end of follow-up, mL/min/1.73 m^2^, median (Q1–Q3)	51.0 (34.5–64.5)	54.0 (45.0–62.0)	23.5 (17.8–36.3)	40.0 (32.0–50.0)	0.0188
Proteinuria at the end of follow-up ≥50 mg/dL, n (%)	2 (2.5)	1 (12.5)	3 (42.9)	0	0.0001
Proteinuria at the end of follow-up, median (Q1–Q3)	0 (0–0)	0 (0–0)	0 (0–51.5)	0 (0–0)	0.2471
>30% decline in eGFR, n (%)	8 (10.0)	1 (11.1)	6 (66.7)	5 (50.0)	<0.0001
Death-censored graft loss, n (%)	1 (1.3)	0	2 (22.2)	1 (10.0)	0.0095
Combined endpoint: >30% decline in eGFR or graft loss, n (%)	9 (11.3)	1 (11.1)	6 (66.7)	5 (50.0)	<0.0001
Time from biopsy to combined endpoint, months, mean ± SD	25.3 ± 9.05	40	19.5 ± 10.03	28.2 ± 12.60	0.2714
Death event, n (%)	4 (5.0)	0	2 (22.2)	0	0.1151

Abbreviations: HLA—human leukocyte antigen; DSAs—donor-specific antibodies; Q1–Q3—quartile 1–3; eGFR—estimated glomerular filtration rate; and SD—standard deviation.

**Table 5:** Univariate Cox proportional hazards model for the combined endpoint according to the evolution of preformed anti-HLA DSAs and development of de novo anti-HLA DSAs at the time of biopsy.

DSA Status	Hazard Ratio	95% Confidence Interval	*p*-Value
No anti-HLA DSAs	Ref.		
Resolved preformed anti-HLA DSAs	1.07	0.136–8.481	0.9470
Persistent preformed anti-HLA DSAs	8.18	2.902–23.03	<0.0001
De novo anti-HLA DSAs	4.61	1.541–13.793	0.0063

Abbreviations: HLA—human leukocyte antigen; DSAs—donor-specific antibodies.

**Table 6:** Multivariate Cox proportional hazards model for the combined endpoint. To build the model, a univariate analysis was undertaken, and significant factors were entered into the multivariate model. A backward stepwise elimination method was used.

Variable	Hazard Ratio	95% Confidence Interval	*p*-Value
Donor’s age (per 1-year increase)	1.03	0.999–1.066	0.0530
Proteinuria at biopsy ≥50 mg/dL (vs. <50 mg/dL)	1.02	1.001–1.036	0.0428
DSA status			
No anti-HLA DSAs	Ref.		
Resolved preformed anti-HLA DSAs	1.10	0.139–8.676	0.9305
Persistent preformed anti-HLA DSAs	5.96	2.041–17.431	0.0011
De novo anti-HLA DSAs	4.48	1.483–13.520	0.0079

Abbreviations: DSA—donor-specific antibody; HLA—human leukocyte antigen.

**Table 7:** Factors associated with DSA status.

Variable	Odds Ratio	95% Confidence Interval	*p*-Value
Resolved preformed anti-HLA DSAs			
Induction therapy (ATG vs. None)	18.25	7.40–44.97	0.0013
Induction therapy (Basiliximab vs. None)	4.56	1.61–12.87	0.1434
Persistent preformed anti-HLA DSAs			
Previous transplantation (yes vs. no)	25.77	10.34–64.23	0.0004
Donor’s age (per 1-year increase)	1.08	1.04–1.13	0.0424
De novo anti-HLA DSAs			
Previous transplantation (yes vs. no)	31.64	13.48–74.23	<0.0001

Abbreviations: HLA—human leukocyte antigen; DSAs—donor-specific antibodies; and ATG—anti-thymocyte globulin.

## Data Availability

The data presented in this study are available on request from the corresponding author.
